# g-Jitter Mixed Convective Slip Flow of Nanofluid past a Permeable Stretching Sheet Embedded in a Darcian Porous Media with Variable Viscosity

**DOI:** 10.1371/journal.pone.0099384

**Published:** 2014-06-13

**Authors:** Mohammed J. Uddin, Waqar A. Khan, Norsarahaida S. Amin

**Affiliations:** 1 Department of Mathematics, American International University- Bangladesh, Banani, Dhaka, Bangladesh; 2 Department of Engineering Sciences, PN Engineering College, National University of Sciences and Technology, Karachi, Pakistan; 3 Department of Mathematical Sciences, Faculty of Science, Universiti Teknologi Malaysia, Johor, Malaysia; Texas A&M University, United States of America

## Abstract

The unsteady two-dimensional laminar g-Jitter mixed convective boundary layer flow of Cu-water and Al_2_O_3_-water nanofluids past a permeable stretching sheet in a Darcian porous is studied by using an implicit finite difference numerical method with quasi-linearization technique. It is assumed that the plate is subjected to velocity and thermal slip boundary conditions. We have considered temperature dependent viscosity. The governing boundary layer equations are converted into non-similar equations using suitable transformations, before being solved numerically. The transport equations have been shown to be controlled by a number of parameters including viscosity parameter, Darcy number, nanoparticle volume fraction, Prandtl number, velocity slip, thermal slip, suction/injection and mixed convection parameters. The dimensionless velocity and temperature profiles as well as friction factor and heat transfer rates are presented graphically and discussed. It is found that the velocity reduces with velocity slip parameter for both nanofluids for fluid with both constant and variable properties. It is further found that the skin friction decreases with both Darcy number and momentum slip parameter while it increases with viscosity variation parameter. The surface temperature increases as the dimensionless time increases for both nanofluids. Nusselt numbers increase with mixed convection parameter and Darcy numbers and decreases with the momentum slip. Excellent agreement is found between the numerical results of the present paper with published results.

## Introduction

The presence of temperature gradient and gravitational field yields convective flows in clear as well as porous media. This type of flow has a significant effect on the homogenous melt growth of semiconductor or metal crystals on earth-bound conditions. In space, the gravity effect is suppressed remarkably and hence buoyancy effect also reduces. However, microgravity environment is helpful in suppressing convective flows. The effect of g-Jitter which originates from crew motions, mechanical vibrations (pumps, motors, excitations of natural frequencies of spacecraft structure), spacecraft maneuvers, atmospheric drag and the earth's gravity gradient (Li [1]) have shown to make it difficult to realize a diffusion-controlled growth from melts in microgravity (Lehoczky [2]). This adverse effect of g-jitter has been the subject of many investigations (see, for instance, Chen and Saghir [3]). A number of researchers studied g-Jitter convective flow in various aspects. Wasu and Rajvanshi [4] studied unsteady mixed convection flow under the influence of gravity modulation and magnetic field. The g-Jitter effects on viscous fluid flow and porous medium have also been investigated by Rees and Pop [5], [6], Chen and Chen [7].

Transport phenomena associated with nanofluids flow have received attention of many investigators due to their diverse applications in many fields such as the delivery of nanodrug (Kleinstreuer et al. [8]), thermal management (Nkurikiyimfura et al. [9]), solar collectors (Said et al. [10]), transportation, the environment and national security (Varjavalu et al. [11] and Wong and Leon [12]). According to Krajnik et al. [13], nanofluids can be optimized during manufacture using sheet processing. Many superior lubricants as well as thermal working fluids may be developed for applications in aerospace, energy systems, medical engineering etc. Available mathematical models for boundary layer flow of nanofluids are (i) Buongiorno [14] model which incorporates Brownian motion and thermophoresis effects and (ii) the Tiwari and Das model [15] which can be used to analyze the behavior of nanofluids taking into account the solid volume fraction. Several authors have used these two models to study various flow phenomena past various types of geometries subject to various initial and boundary conditions. As an example, Kuznetsov and Nield [16] obtained similarity solution for natural convection flow of a nanofluid along a vertical plate. The effects of Brownian motion and thermophoresis on boundary layer flow of nanofluids past a flat surface in a porous medium was studied by Nield and Kuznetsov [17]. Chamkha et al. [18] investigated the natural convective flow of nanofluid past an isothermal sphere placed in a Darcy porous medium. Khan and Aziz [19] studied the double-diffusive natural convection from a vertical plate to a porous medium saturated with a binary base fluid containing nanoparticles. Analytical and numerical analysis of heat and mass transfer of nanofluid was carried out by Kameswaran et al. [20]. Finite difference solution of transport equations due to nanonofluid flow past a vertical plate taking into account double stratification was presented by Ibrahim and Makinde [21]. Cimpean and Pop [22] investigated nanofluid mixed convection in a porous medium channel. Nanofluid convection flows in porous media have received attention motivated by materials processing and solar energy collector applications. Gorla et al. [23] used a Blottner difference method to simulate mixed convection flow from a vertical wedge in a porous medium saturated with a nanofluid. Bég et al. [24] used finite difference numerical method to study nanofluid flow, heat and mass transfer in a porous medium. Further studies of nanofluid convection transport in porous media have been reported by Gorla and Chamkha [25] for non-isothermal effects, Yasin et al. [26] for heat generation effects, Kuznetsov [27] for non-oscillatory and oscillatory bioconvection, Murthy et al. [28] for magnetic effect on thermally stratified medium. Bég et al. [29] used a homotopy analysis method to simulate nanofluid free convection from a spherical body in a Darcian porous medium. Chamkha and Rashad [30] studied steady laminar natural convection boundary layer flow over a permeable vertical cone in a porous medium saturated with a nanofluid. Very recently, Uddin et al. [31], present a mathematical model of nonlinear radiative hydromagnetic thermo-solutal nanofluid convection slip flow in saturated porous media. Relatively few studies of flow with multiple slip effect in porous media have been communicated.

Much research has been focused on heat and fluid flow at micro-scale and nano-scales because of the applications in micro-electro-mechanical systems and nano-electro-mechanical systems with the inclusion of velocity and temperature slip boundary conditions at the wall, as for this type of flow convectional no slip conditions yield unrealistic results. In the slip region, the Navier–Stokes and energy equations can still be used provided the velocity slip and temperature slip at the walls are taken into account simultaneously (Karniadakis et al. [32]). Bocquet and Barrat [33] illustrated the mechanisms of surface slip (from nano to micro scales) and heat transfer on the interface. Various aspect of fluid flow and heat transfer of nanofluid slip flows past various geometries were investigated by many authors (Robert et al. [34], Wang [35], Zheng [36], Wu [37], Noghrehabadi et al. [38], Matthews and Hill [39], Das [40], Bhattacharyya et al. [41]).

It seems that most of the published papers on nanofluid considered viscosity as constant. However, in reality it depends on temperature. Heat generated by internal friction and the corresponding rise in the temperature affects the viscosity of the fluid, so that the fluid viscosity no longer is considered constant (Mukhopadhyay and Layek [42]). Prasad et al. [43] study the effect of variable fluid properties on the hydromagnetic flow and heat transfer over a stretching sheet. The effect of temperature dependent viscosity and thermal conductivity on flow, heat and mass transfer flow was investigated by Hamad et al. [44]. Pal and Mondal [45] investigated the effect of variable viscosity on mixed convection flow and heat transfer along a stretching sheet a in a non-Darcy porous medium. Hence, in order to get the real picture of the flow, heat and mass transfer characteristics in the nanofluids, it is required to take into the effect of the temperature-dependent viscosity of the base fluid. This motivates the present study.

In the present work, we investigate the effects of linear hydrodynamic slip, thermal slip and temperature dependent viscosity on g-Jitter mixed convective boundary layer flow of nanofluid past a permeable stretching sheet in a Darcian porous media. The governing boundary layer equations are transformed to non-similar equations using suitable transformations. Numerical solutions are obtained using an implicit finite difference method. Validation with published paper is achieved. The effect of the controlling parameters on the dimensionless velocity, temperature, friction factor and heat transfer rates are illustrated via figures.

## Nanofluid Dynamic Transport Model

**Figure 1:**
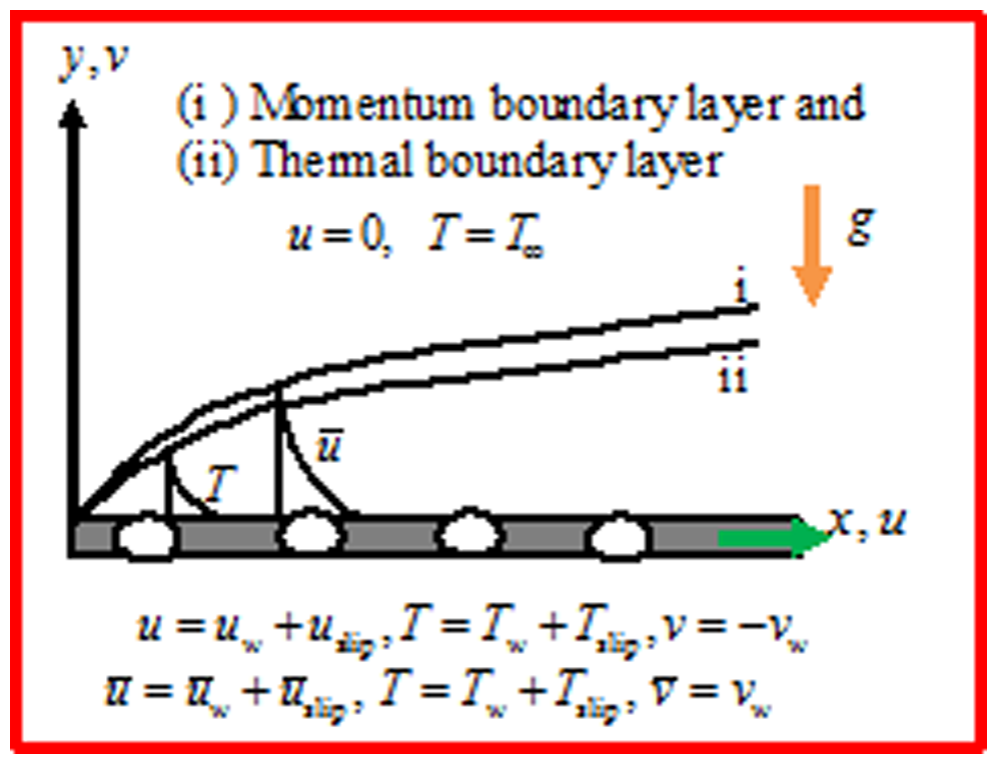
Geometry of the flow model and coordinate system.



Consider the steady 2-D laminar boundary layer flow of viscous water-based nanofluids containing Cu and Al_2_O_3_ nanoparticles. The base fluid and the nanoparticles are assumed to be in thermal equilibrium. It is assumed that the plate is moving with a velocity 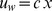 in the quiescent free stream. It is further assumed that the plate surface is subjected to linear hydrodynamic and thermal slip boundary conditions. We consider the nanofluid is a two component mixture with the following assumptions: (i) incompressible; (ii) no-chemical reaction; (iii) negligible viscous dissipation; and (v) nano-solid-particles and the base fluid are in thermal equilibrium. The effect of g-jitter is induced by mixed convective flow of a nanofluid past a permeable vertical stretching. Following Sharidan et al. [46], the gravity acceleration is given by 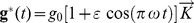, where 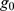 is the time-averaged value of the gravitational acceleration, 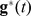 acting along the direction on the unit vector 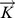, which is oriented in the upward direction, 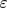 is a scaling parameter, which gives the magnitude of the gravity modulation relative to 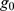, 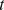 is the time and 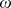 is the frequency of oscillation of the g-Jitter driven flow. If 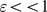 then the forcing may be seen as a perturbation of the mean gravity. The scheme of physical configuration is shown in **Fig.1**. Field variables are Darcian tangential and normal velocity components *u*, v and temperature 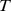. Under these assumptions, the basic continuity, momentum and energy equations in dimensional form can be written as (Tiwari and Das [15], Vajravalu et al. [47])

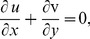
(1)

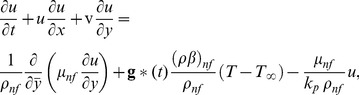
(2)

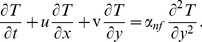
(3)


The initial and boundary conditions are (Karniadakis et al. [32])
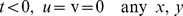


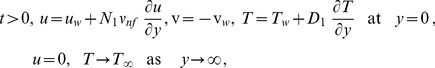
(4)where 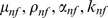 are the properties of nanofluids and are given by (Oztop and Abu-Nada [48])



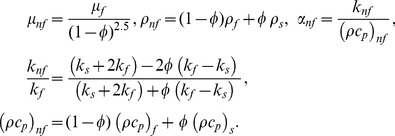
(5)Here 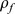 is the density, 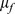 is the viscosity, 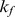 is the thermal conductivity of the base fluid, 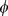 is the solid volume fraction parameter of the nanofluid (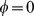 corresponds to clear fluid), 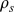 is the the density of the nanoaprticles, 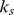 is the thermal conductivity of the nanoparticles, 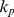 is the permeability of the porous media, 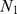 is the velocity slip factor having dimensions of 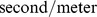, 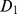 is the thermal slip factor having dimensions of 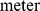, 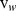 is the mass transfer velocity, 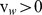 is for suction and 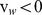 for injection. Further, 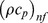 is the heat capacitance of the nanofluid, 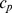 is the specific heat at constant pressure. Following Vajravalue et al. [47], the thermal expansion coefficient of the nanofluid can be determined by 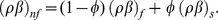 Following Mukhopadhyay and Layek [49], Hamad et al. [44] we assume the temperature dependent viscosity varies according to 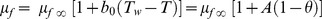, where 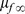 is the constant undisturbed viscosity 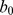 is constant with 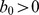, 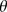 is defined in Eqn.(6), 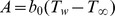 is the viscosity variation parameter.

To reduce the number of dependent variables as well as number of equations, we use stream function 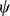 defined by 
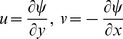
. Note that 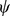 satisfies equation of continuity automatically. Now, following Sharidan et al. (2006), we introduce following transformations 
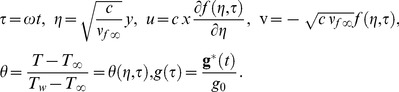
(6)


Substituting Eqn. (6) into Eqns. (2)–(3), we obtain the following differential equations
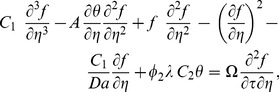
(7)

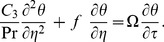
(8)


The boundary conditions (4) become
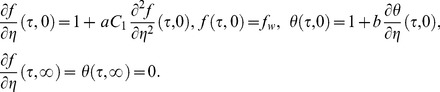
(9)


The constants 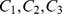 are defined as:
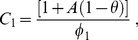
(10)

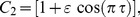
(11)

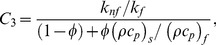
(12)

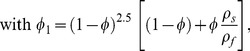
(13)

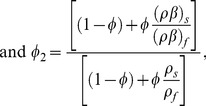
(14)where 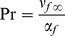 is the Prandtl number, 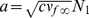 is the velocity slip parameter, 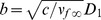 is the thermal slip parameter, 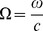 is the non-dimensional frequency, 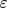 is the amplitude of modulation, 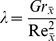 is the mixed convection parameter, 
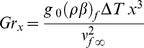
 is the local Grashof number, 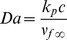 is the Darcy number and 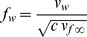 is the suction/injection parameter, 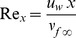 is the local Reynolds number. Thermophysical properties of the base fluid and nanoparticles are reported in Table 1.

**Table 1:** Thermophysical properties of the base fluid and nanoparticles [48].

Physical properties	Base fluid	Nanoparticles	
	Water	Cu	Al_2_O_3_
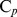 (J/kg-K)	4179	385	765
ρ(kg/m^3^)	997.1	8933	3970
k(W/m-K)	0.613	401	40
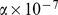 (m^2^/s)	1.47	1163.1	1738.6

For the first level of truncation (or steady state), the time derivatives in Eqns. (7)–(8) can be neglected. Thus the steady state governing equations for the first level of the truncation can be written as
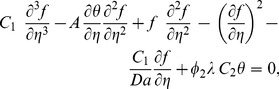
(15)

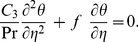
(16)


The boundary conditions (9) become
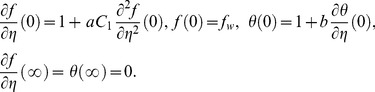
(17)


The steady state solution of Eqns. (15) and (16) with boundary conditions (17) is found numerically and compared with Grubka and Bobba [50] in Table 2.

**Table 2:** Comparison of dimensionless heat transfer rates 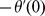 for different values of Pr when 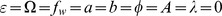.

Pr	Grubka and Bobba [50]	Present Results
0.72	0.4631	0.46325
1	0.5820	0.58198
3	1.1652	1.16524
10	2.3080	2.30800
100	7.7657	7.76565

Assuming new functions 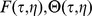 which are defined by
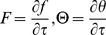
(18)and restoring all of the terms neglected in the first level of truncation, the governing equations can be written as



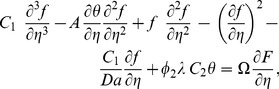
(19)

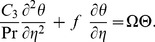
(20)


The boundary conditions (4) become
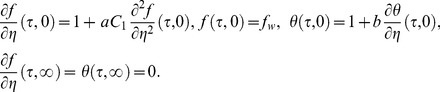
(21)


Eqns. (19) and (20) contain two additional functions. In order to solve these equations, we need two more equations which can be obtained by differentiating Eqns. (19)–(20) with respect to 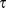. Neglecting derivatives of 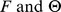 with respect to 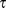, and simplifying, we get
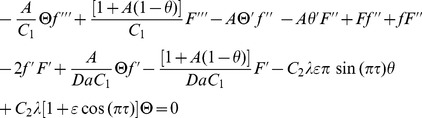
(22)

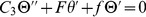
(23)with new boundary conditions
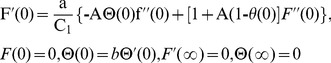
(24)


## Quantities of Physical Interest

The quantities of engineering interest, in this study, are the local skin friction factor 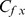 and the local Nusselt number 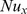 can be found from the following definition 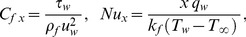
(25)where 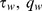 are shear stress and the wall heat flux (dimensional) and are defined as



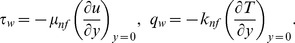
(26)Using Eqns. (6) and (26), we have from Eqn. (25)
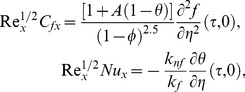
(27)where 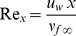 is the local Reynolds number.

## Validation with Published Results

It is interesting to note that if we put 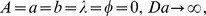 in Eqns. (15)–(17), we have the same Eqns. as derived by Grubka and Bobba [50]. A comparison of 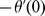 is presented in Table 2 for the special case of steady-state flow (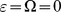), no suction/injection (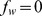), no slip (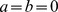), conventional fluid (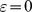) and constant viscosity (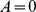). The present numerical results are found to be in good agreement for each value of Pr. We, therefore, are confident that our results are accurate.

## Results and Discussions

**Figure 2:**
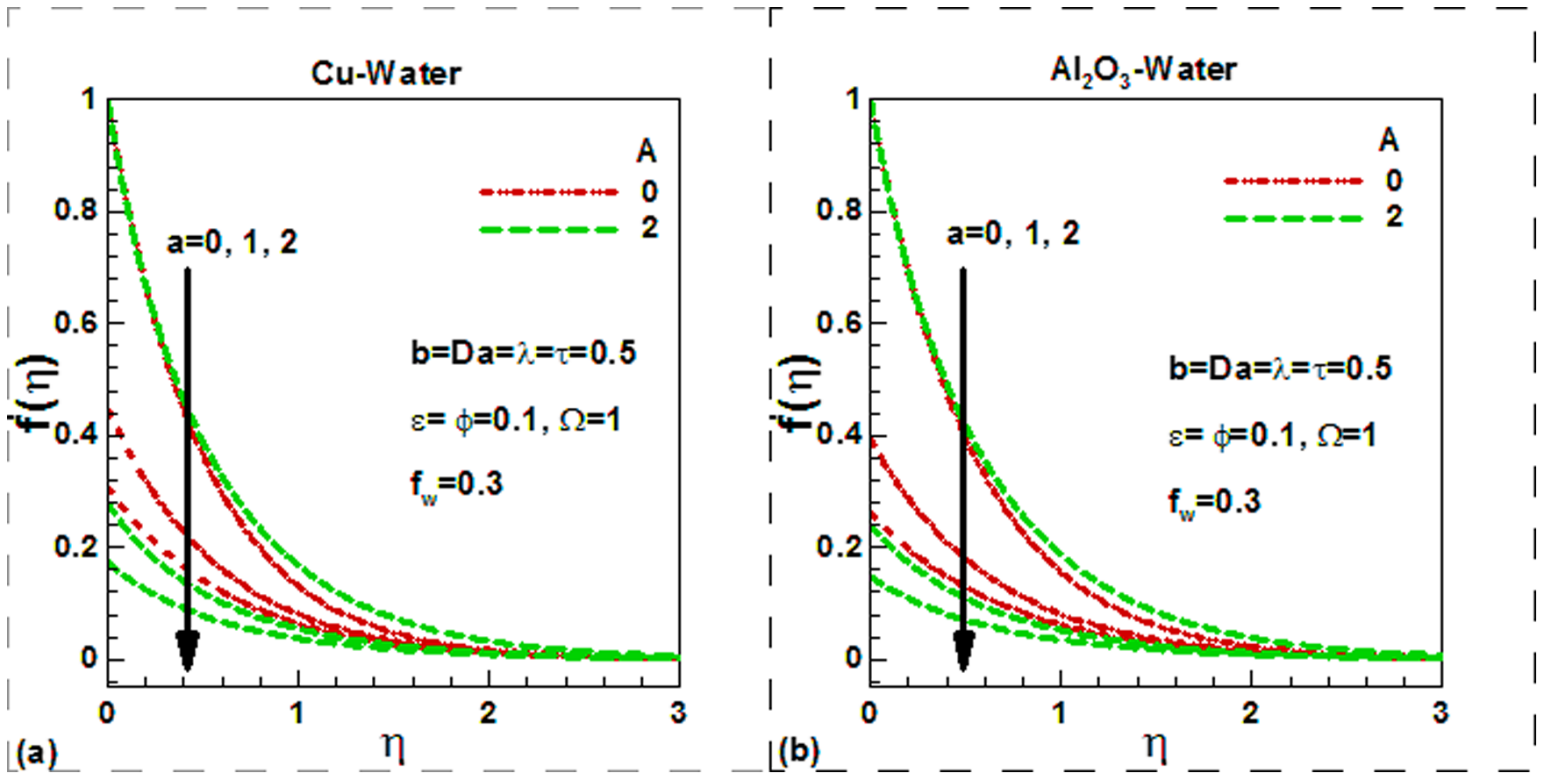
Effects of viscosity variation and velocity slip parameters on dimensionless velocity for (a) Cu-water and (b) Al_2_O_3_-water nanofluids.

**Figure 3:**
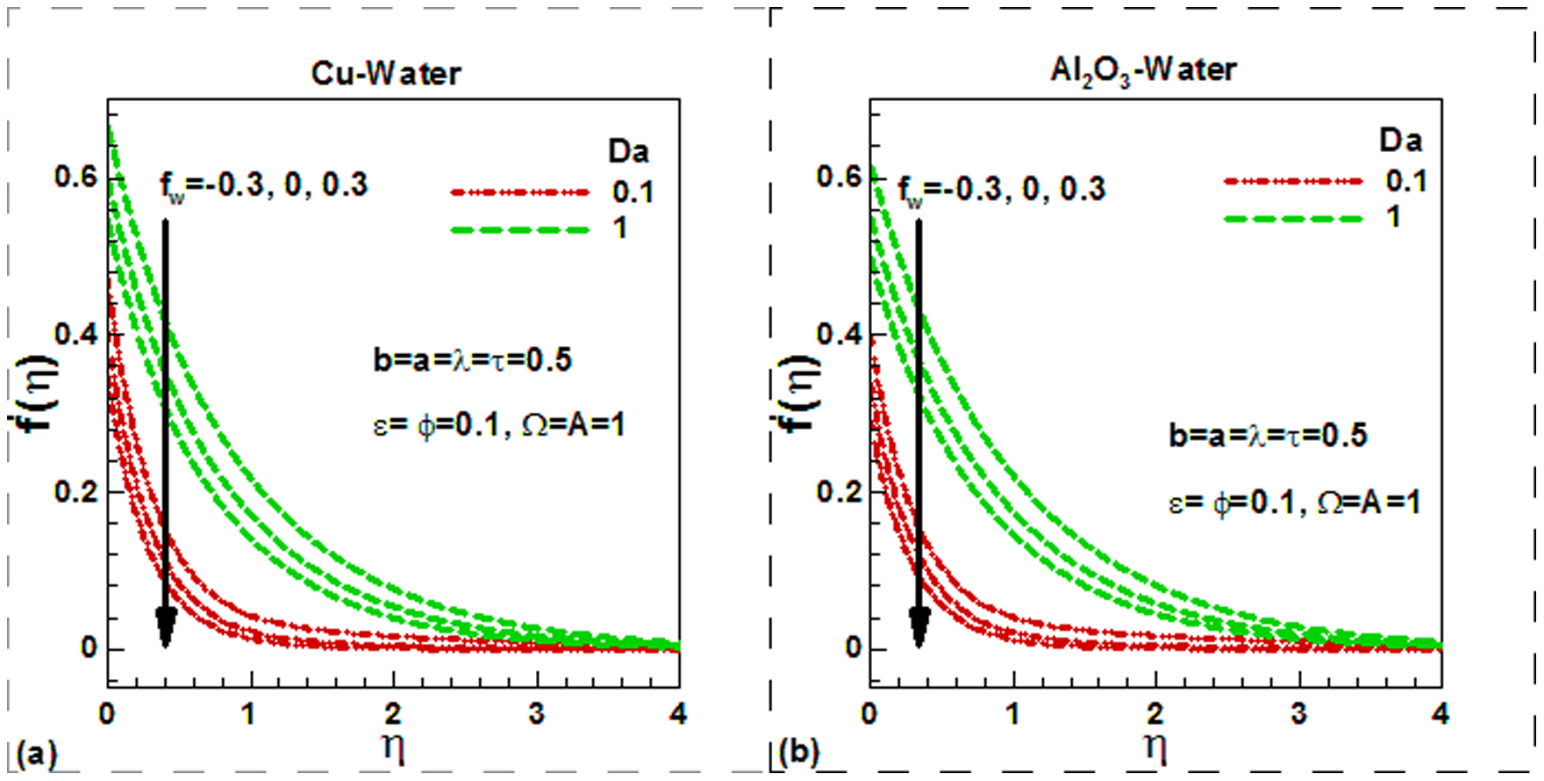
Effects of Darcy number and suction/injection parameters on dimensionless velocity for (a) Cu-water and (b) Al_2_O_3_-water nanofluids.

**Figure 4:**
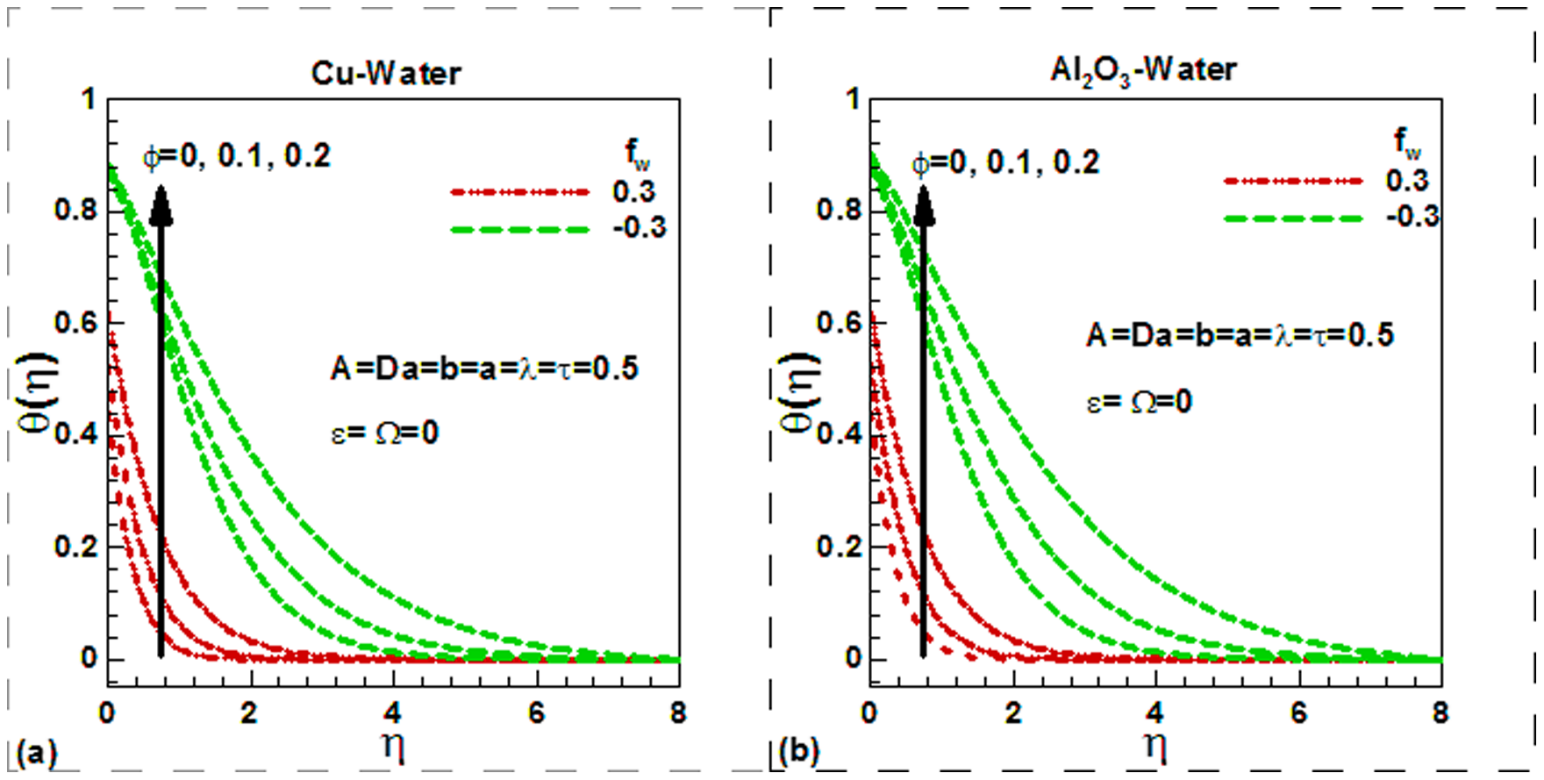
Effects of suction/injection parameters and solid volume fraction of nanoparticles on dimensionless temperature for (a) Cu-water and (b) Al_2_O_3_-water nanofluids.

**Figure 5:**
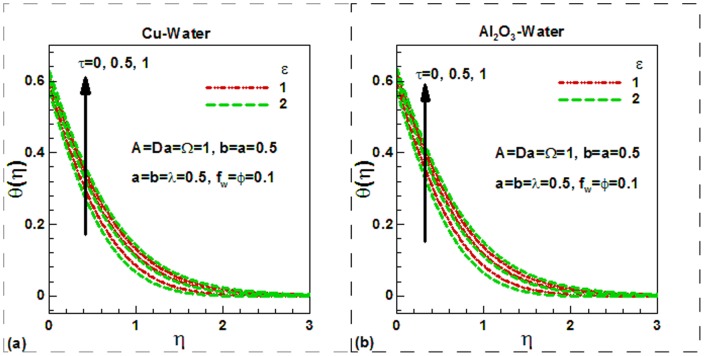
Effects of dimensionless time and scaling parameters on dimensionless temperature for (a) Cu-water and (b) Al_2_O_3_-water nanoflu.

**Figure 6:**
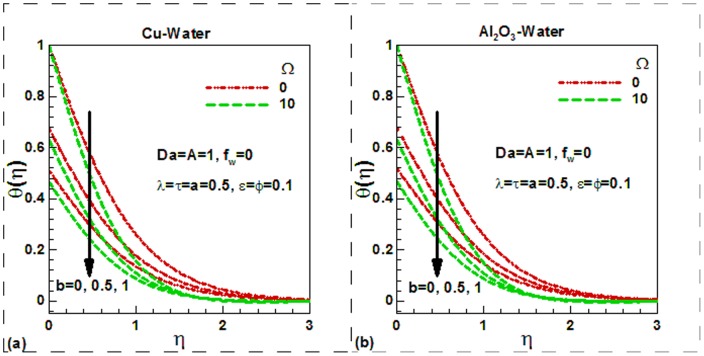
Effects of thermal slip and dimensionless frequency on dimensionless temperature for (a) Cu-water and (b) Al_2_O_3_-water nanofluids.

**Figure 7:**
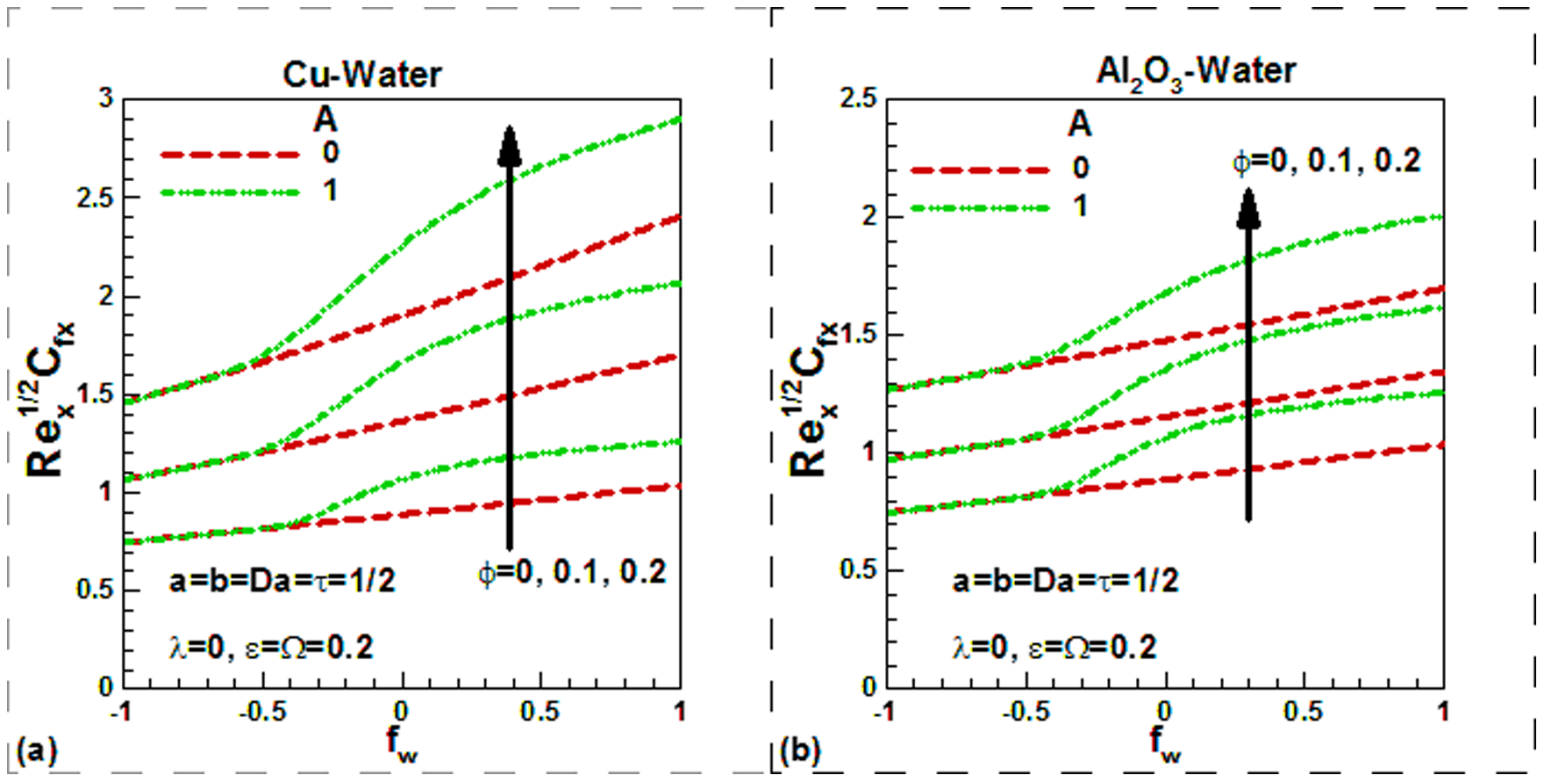
Variation of skin friction with suction/injection parameters and solid volume fraction of nanoparticles for different values of viscosity variation parameter for (a) Cu-water and (b) Al_2_O_3_-water nanofluids.

**Figure 8:**
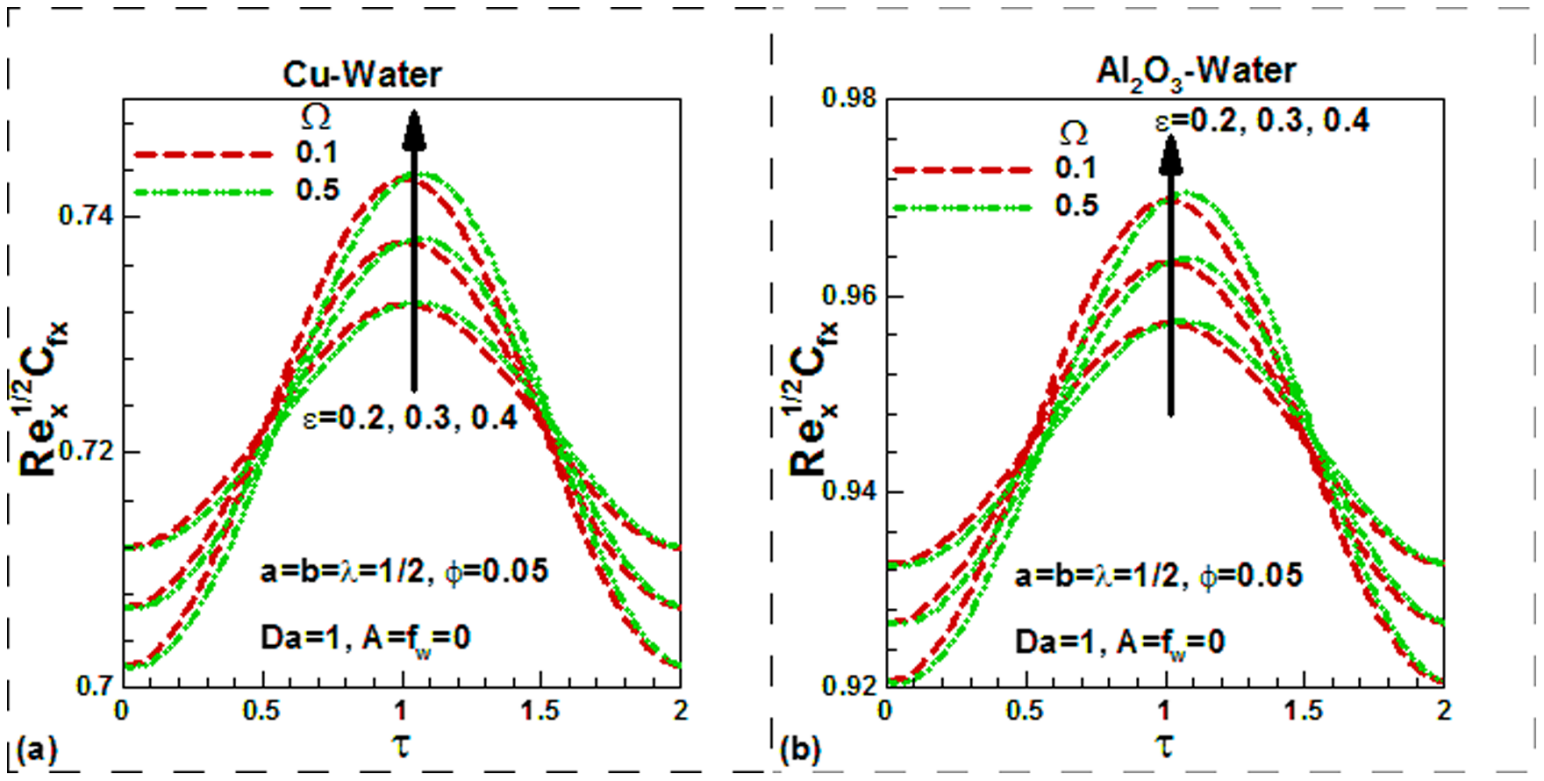
Variation of skin friction with dimensionless time and scaling parameters for different values of dimensionless frequency for (a) Cu-water and (b) Al_2_O_3_-water nanofluids.

**Figure 9:**
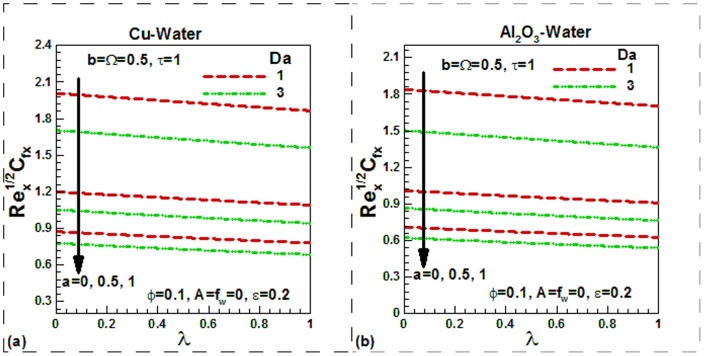
Variation of skin friction with mixed convection and velocity slip parameters for different values of Darcy number for (a) Cu-water and (b) Al_2_O_3_-water nanofluids.

**Figure 10:**
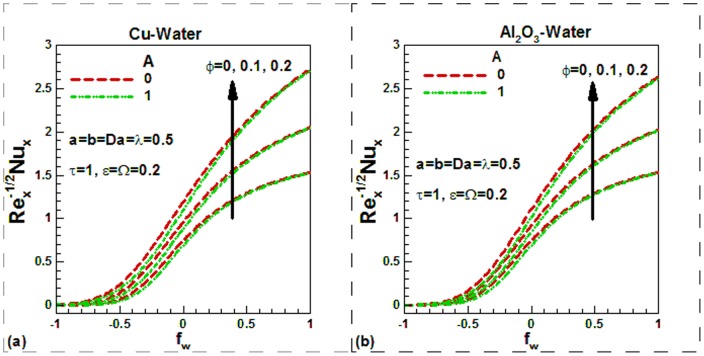
Variation of Nusselt numbers with suction/injection parameters and solid volume fraction of nanoparticles for different values of viscosity variation parameter for (a) Cu-water and (b) Al_2_O_3_-water nanofluids.

**Figure 11:**
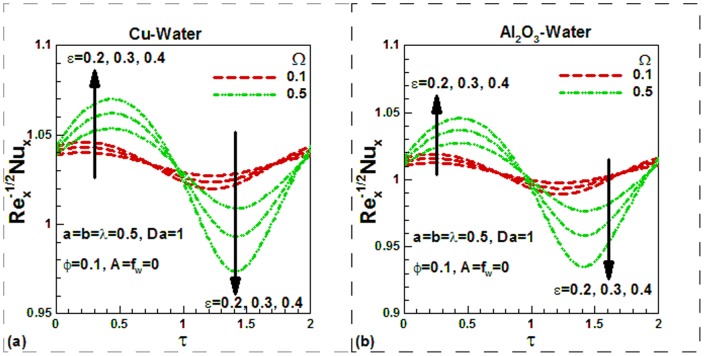
Variation of Nusselt numbers with dimensionless time and scaling parameters for different values of dimensionless frequency for (a) Cu-water and (b) Al_2_O_3_-water nanofluids.

**Figure 12:**
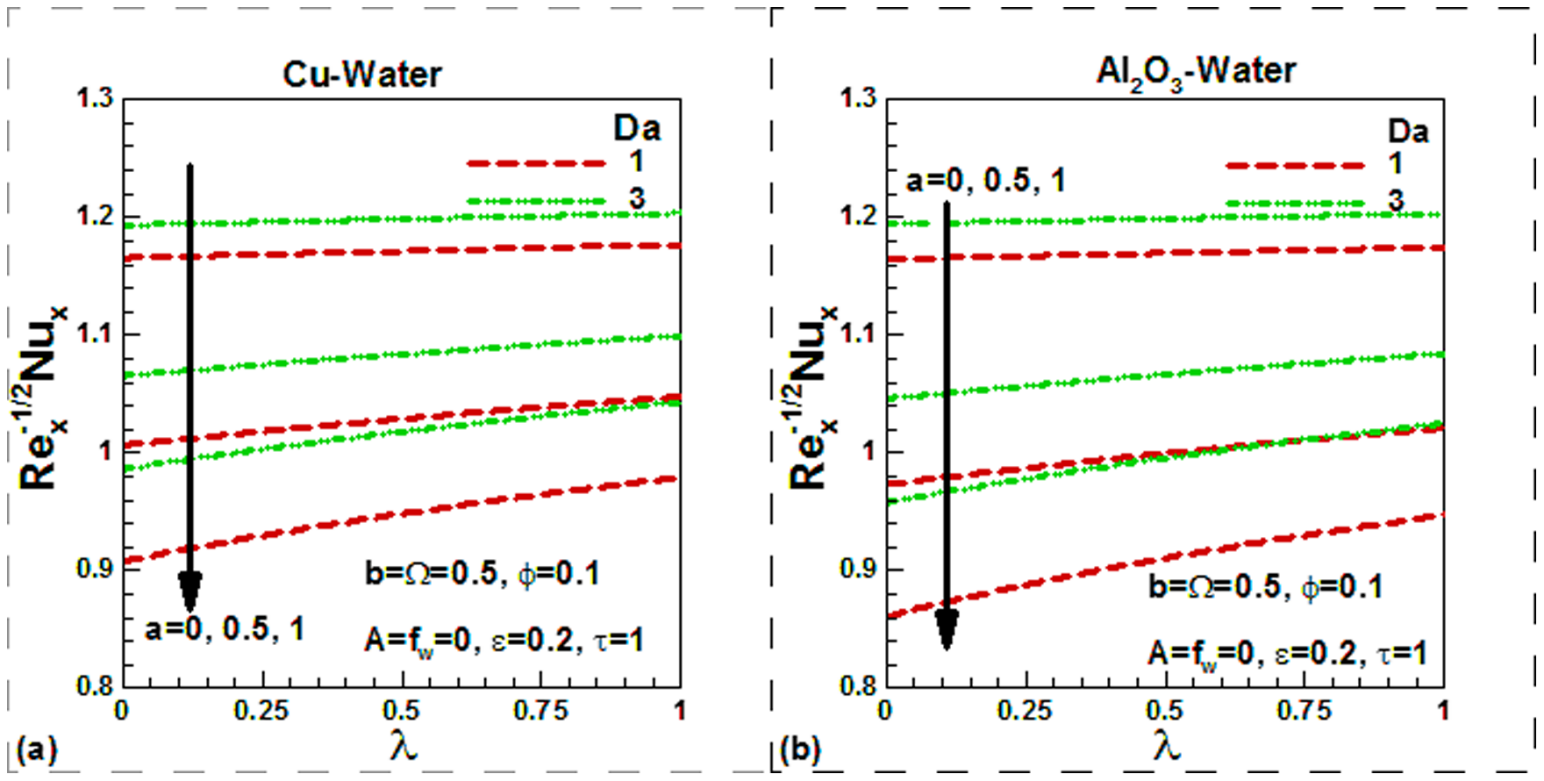
Variation of Nusselt numbers with mixed convection and velocity slip parameters for different values of Darcy number for (a) Cu-water and (b) Al_2_O_3_-water nanofluids.

Equations (19), (20), (22) and (23) with boundary conditions (21) and (24) were solved numerically to obtain the dimensionless velocity and temperature fields as well as the skin friction and heat transfer rate. The effects of viscosity variation and velocity slip parameters on the dimensionless velocity are compared for Cu-water and Al_2_O_3_-water nanofluids in Figs. 2(a) and 2(b). The other parameters are kept fixed and suction is considered in this case. For uniform viscosity and no slip condition, the dimensionless surface velocity of both nanofluids is found to be higher in unsteady flow. But as slip parameter increases, the dimensionless velocity at the surface decreases and converges quickly. As a result, the hydrodynamic boundary layer thickness and hence the skin friction decreases. Physically, as the velocity slipping parameter 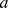 increases the differences between the wall and the fluid velocities near to the wall rises. Increasing the slipping factor may be looked at as a miscommunication between the source of motion (the plate) and the fluid domain. Note that the case a = 0.0 corresponds to conventional no slip boundary conditions. It is noticed from the same figure that the dimensionless velocity increases with an increase in viscosity parameter for both in the case of slip and no slip boundary conditions. Physically, with an increase in the viscosity parameter A, fluid viscosity decreases resulting the increment of velocity boundary layer thickness. Due to lower density of Al_2_O_3_ nanoparticles, the dimensionless velocity of Al_2_O_3_-water nanofluids is found to be smaller at the surface. The Darcy number, in fact, shows the ability of fluids to flow through porous medium. The greater the Darcy number the greater will be the flow of fluids and hence the greater velocity of fluids. This is illustrated in Figs. 3(a) for Cu-water and in Fig. 3(b) for Al_2_O_3_-water nanofluids respectively. The suction velocity tends to reduce the boundary layer thickness whereas the boundary layer thickness increases in case of injection. This is shown in Figs. 3(a) and (b) for both nanofluids. Physically, suction causes the boundary layer to adhere more closely to the wall and this destroys momentum leading to a plummet in velocity. Hydrodynamic boundary layer thickness is therefore decreased with suction. Injection adds nanofluid velocity via lateral mass flux through the sheet and this assists momentum development, enhancing velocity and causing a concomitant increase in momentum boundary layer thickness. Again due to lower density of Al_2_O_3_ nanoparticles, the dimensionless surface velocity of Al_2_O_3_-water nanofluids is found to be lower. The effects of different controlling parameters on the dimensionless temperature are shown in Figs. 4–6 for both Cu-water and Al_2_O_3_-water nanofluids. The effects of solid volume fraction of nanoparticles on the dimensionless temperature are shown in Figs. 4(a) and (b) for suction and injection of both nanofluids. In unsteady flows, the solid volume fraction of nanoparticles increases the dimensionless temperature of both nanofluids inside the thermal boundary layer and as a result the thermal boundary layer thickness increases for the selected values of the controlling parameters. The suction/injection parameters have strong effects on the thermal boundary layer thickness. In case of suction, the thermal boundary layer thickness is found to be smaller than in injection for both nanofluids. However, due to higher thermal conductivity of Cu-water nanofluids, the dimensionless surface temperature of Cu-water nanofluids is found to be lower than Al_2_O_3_-water nanofluids. The effects of the dimensionless time and the scaling parameter on the dimensionless temperature are depicted in Figs. 5(a) and (b) for both nanofluids. The scaling parameter 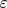 shows the magnitude of the gravity modulation relative to 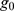. It can be seen that for steady case, when 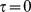, the surface temperature is found to be lower for smaller values of 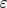 in both cases. As the dimensionless time increases, the surface temperature increases for both nanofluids. However, both the dimensionless time and the scaling parameter have negligible effect on the thermal boundary layer thickness. The variation of the dimensionless temperature with the transverse distance inside the thermal boundary layer is shown in Figs. 6(a) and (b) for both nanofluids. The effects of thermal slip and dimensionless frequency are investigated while all the other parameters are kept constant in both cases. It can be seen that, in the absence of thermal slip, the surface temperature is highest and it reduces with an increase in the thermal slip. In steady-state (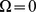), the dimensionless temperature is found to be higher in the thermal boundary layer in both cases. The variation of skin friction with different controlling parameters is shown in Figs. 7–9 for the selected nanofluids. The skin friction is found to increase in case of suction and decrease in case of injection as shown in Fig. 7(a) for Cu-water and in Fig. 7(b) for Al_2_O_3_-water nanofluids respectively. The effects of temperature dependent viscosity on skin friction can also be observed in these figures. When the viscosity is constant, the skin friction is found to be lower and it increases with temperature. It is important to note that as we go from suction to injection region, the effects of viscosity variation parameter diminish. In the absence of nanoparticles (pure water), the skin friction is found to be lowest. But as the solid volume fraction of nanoparticles increases, the skin friction increases due to higher density of nanofluids. Since the density of Cu-water nanofluids is higher than Al_2_O_3_-water nanofluids, the skin friction for Cu-water nanofluids is found to be higher. Due to sinusoidal nature, the skin friction first increases up to maximum value and then decreases with dimensionless time. This is shown in Figs. 8(a) and (b) for two selected water-based nanofluids when there is no suction or injection. It can be seen that the scaling parameter has no appreciable effect on the skin friction in any case. The variation of skin friction with mixed convection parameter 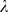 for different values of Darcy number and momentum slip is shown in Figs. 9(a) and (b) for two selected nanofluids. In case of forced convection (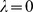), the skin friction is found to be higher than in free convection (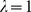) in both case. Both Darcy number and momentum slip tend to reduce skin friction in both cases. Figures 10–12 show the variation of Nusselt numbers with different controlling parameters for two selected water-based nanofluids. It can be seen that the Nusselt numbers for pure water (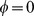) are lower and increase with an increase in the solid volume fraction of nanoparticles, as shown in Figs. 10 (a) and (b) for both nanofluids. This is due to increase in thermal conductivity with the solid volume fraction of nanoparticles. However, the effects of viscosity variation parameter on the Nusselt numbers are not appreciable. Like skin friction, Nusselt numbers also increase during suction and decrease during injection. The sinusoidal nature of Nusselt numbers can be observed in Figs. 11 (a) and (b) for both nanofluids. The case of uniform viscosity without any suction/injection is considered for investigating the effects of scaling parameter and dimensionless frequency on the Nusselt numbers for both nanofluids. The effects of mixed convection on the Nusselt numbers for different values of Darcy number and momentum slip are shown in Figs. 12(a) and (b) for both nanofluids. As expected, the Nusselt numbers increase with mixed convection parameter and Darcy numbers. But this increase is found to be negligible in no slip condition. As the momentum slip increases, Nusselt numbers decrease. Since the thermal conductivity of Cu is higher than Al_2_O_3_, the Nusselt numbers are found to be higher for Cu-water nanofluids.

## Conclusion

The two-dimensional unsteady laminar g-jitter mixed convective boundary layer flow of Cu-water and Al_2_O_3_-water nanofluids past a permeable stretching sheet in a Darcian porous is studied by combined non-similar and numerical solution technique. Main findings are given below.

1. Dimensionless velocity reduces as velocity slip parameter increases for both nanofluids and for fluid with constant and variable properties.
2. Dimensionless velocity increases as Darcy numbers increase for both nanofluids in the case of both permeable and impermeable plate.
3. Dimensionless surface temperature increases as nanoparticle volume fraction increases for both nanofluids
4. Dimensionless surface temperature increases as the dimensionless time increases for both nanofluids
5. Dimensionless surface temperature reduces with an increase of the thermal slip.
6. The skin friction increases with as the viscosity parameter increases.
7. Both Darcy number and momentum slip tend to reduce skin friction for both nanofluid.
8. Nusselt numbers increase with Darcy numbers and decrease with the momentum slip.
